# Mass Spectrometry Imaging for Spatial Ingredient Classification in Plant-Based Food

**DOI:** 10.1021/jasms.4c00353

**Published:** 2024-12-07

**Authors:** Mudita Vats, Bryn Flinders, Theodoros Visvikis, Corinna Dawid, Thomas F. Hofmann, Eva Cuypers, Ron M. A. Heeren

**Affiliations:** † Maastricht MultiModal Molecular Imaging (M4I) Institute, Division of Imaging Mass Spectrometry, Maastricht University, Universiteitssingel 50, 6229 ER Maastricht, The Netherlands; ‡ Chair of Food Chemistry and Molecular and Sensory Science, Technical University of Munich, Lise-Meitner-Str. 34, Freising 85354, Germany; § Professorship for Functional Phytometabolomics, TUM School of Life Sciences, Technical University of Munich, Lise-Meitner-Str. 34, Freising 85354, Germany; ∥ Focus Group Molecular Imaging of Cellular Metabolism, Institute for Advanced Studies, Technical University of Munich, Lichtenbergstraße 2a, 85748 Garching, Germany

## Abstract

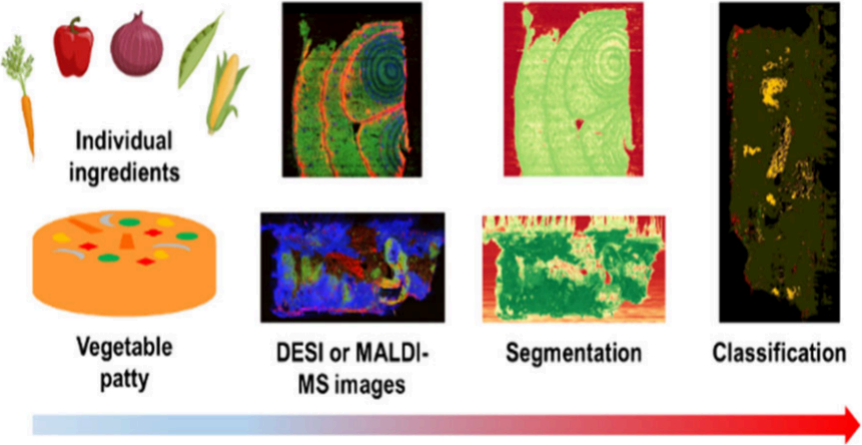

Mass spectrometry imaging (MSI) techniques enable the generation of molecular maps from complex and heterogeneous matrices. A burger patty, whether plant-based or meat-based, represents one such complex matrix where studying the spatial distribution of components can unveil crucial features relevant to the consumer experience or production process. Furthermore, the MSI data can aid in the classification of ingredients and composition. Thin sections of different burger samples and vegetable constituents (carrot, pea, pepper, onion, and corn) were prepared for matrix-assisted laser desorption/ionization (MALDI) and desorption electrospray ionization (DESI) MSI analysis. MSI measurements were performed on all samples, and the data sets were processed to build three machine learning models aimed at detecting meat adulteration in vegetable burger samples, identifying individual ingredients within the vegetable burger matrix, and discriminating between burgers from different manufacturers. Ultimately, the successful detection of adulteration and differentiation of various burger recipes and their constituent ingredients were achieved. This study demonstrates the potential of MSI coupled with building machine learning models to enable the comprehensive characterization of burgers, addressing critical concerns for both the food industry and consumers.

## Introduction

Food is composed of micro- and macronutrients, which makes it a complex matrix on the molecular level. These molecules form specific arrangements that lead to unique chemical and structural properties. Unravelling this complexity is crucial to meet consumer demands for various reasons. LC-MS (liquid chromatography–mass spectrometry) and GC-MS (liquid chromatography–mass spectrometry) provide information with high accuracy and sensitivity and identify a wide range of molecules. They are widely used in food analysis, in the study of food fraud, authenticity, origin traceability and food safety.^1−3^ However, chromatography-coupled MS requires homogenization and extraction of samples and thus lacks information about the spatial distribution of the ingredients.

Spatial information plays a pivotal role in unraveling the complexity of a food matrix. These complexities are linked to the desirable characteristics of food.^4^ MSI (mass spectrometry imaging) techniques such as matrix-assisted laser desorption/ionization (MALDI), secondary ion mass spectrometry (SIMS), laser-assisted rapid evaporative ionization mass spectrometry (LA-REIMS), desorption electrospray ionization (DESI), and laser ablation electrospray ionization (LAESI) have gained research popularity in agricultural and food chemistry. They have been utilized to describe the spatial distribution of sugars, organic acids, lipids, defense related metabolites and pesticides in fruits, vegetables, and crops.^5−9^ MSI can assess the heterogeneity and identify potential variations in the ingredient distributions across different regions of the burger. This information can be valuable in the improvement of ingredient sourcing, processing, and consistent recipe formulation. In contrast to LC-MS studies, which would involve the homogenization and separation of different parts of fruits or vegetables to analyze their molecular composition separately, MSI offers the advantage of simultaneous molecular composition analysis across various parts as well as at the interfaces between structural parts.^10,11^ For example, a cheese section was examined using MALDI-MSI for the penetration and distribution of the preservative natamycin at a spatial resolution of 20 μm.^12^ For food products such as burgers, understanding the spatial distribution and arrangement of various ingredients holds significant importance. This spatial information can facilitate the identification of potential adulterations or the determination of ingredient sources, which is a critical concern for both the food industry and consumers. Recent advancements in mass spectrometry have led to improved analysis in food and agricultural settings. One example is the development of miniaturized portable MS systems, which have been utilized to analyze agrochemicals and environmental toxins in crops and industries.^13,14^ Building on these innovations, the evolution of food analysis using mass spectrometry imaging (MSI) enables spatial ingredient verification in food production processes. This will enhance food quality and safety analysis. Training classification models to recognize and differentiate the various ingredients based on their spatial patterns and spectral signatures can enable automated and objective assessment of burger compositions.

Integrating machine learning models into MSI offers a sophisticated method for following the original ingredients of burgers.^15^ Recognition models based on complex MSI data have been used to classify healthy and diseased tissue and cell types in biomedical research.^16^ Similarly, well-trained models could be utilized to distinguish various components of a mixed food matrix, such as the ingredients in burgers and hamburgers from different manufacturers. A deeper understanding of burger composition can be obtained that provides deeper insights into composition to the consumer by employing these models. Uniform Manifold Approximation and Projection (UMAP) has been successfully applied to MSI data sets, showing great potential in unravelling molecular and spatial complexity.^17,18^

This study investigated both DESI and MALDI-MS imaging techniques to evaluate their complementary nature in the analysis of food products. Initially, the acquired data sets were utilized to distinguish a standard vegetable burger and a purposefully modified vegetable burger containing an added meat component within vegetable burger samples. Subsequently, the scope was expanded to classify and identify the individual ingredients present within the vegetable burger matrix. Ultimately, the study aimed to discriminate between burgers of varying compositions.

## Methods

### Materials

The following samples were purchased from a local supermarket: green peas (*Pisum sativum*), onion (*Allium cepa*), corn (*Zea mays*), red pepper (*Capsicum annuum*), and carrots (*Daucus carota*). Vegetable burger (VB 1) consists of the above ingredients in different quantities; vegetable burger with seeds (VB 2), plant burger (PB) comprised of pea protein, and meat burger (MB) consisting of approximately 90% beef were also used. All of the above burgers also contained vegetable oils, flavoring agents, and seasonings. All samples analyzed were uncooked.

2,5-Dihydroxybenzoic acid (DHB; 98%), and norharmane (NOR; 98% crystalline) were purchased from Sigma-Aldrich (Zwijndrecht, The Netherlands). UPLC-grade acetonitrile (ACN), chloroform (CHCl_3_), methanol (MeOH), and water (H_2_O) were purchased from Biosolve (Valkenswaard, The Netherlands).

### Sample Preparation

The nine different sample types were prepared as follows: (1) frozen green peas were used as purchased, (2) cross sections of carrots were cut into small chunks (1 cm in length), (3) the flesh (pericarp) of the red pepper was cut into 1 cm^2^ squares, (4) a whole onion was first cut transversally and longitudinally to expose the bulb region, (5) single corn kernels were removed from a precooked corn cob, and (6) all the burgers were cut into small cubes with scalpel. All the samples were snap-frozen using liquid nitrogen and stored at −80 °C before analysis.

The frozen samples were sectioned at −20 °C using a Microm HM535 cryomicrotome (Microm International, Walldorf, Germany) to produce 20–30 μm thick sections. The sections were thaw-mounted onto clean indium tin oxide (ITO) coated glass slides (4–8 Ω resistance, Delta Technologies, Loveland, CO, USA) or regular microscope glass slides (Thermo Scientific, Braunschweig, Germany) depending on the instrument used. Later, an intentionally modified vegetable burger was prepared by introducing a meat component. A cavity was made within the vegetable burger (VB 1) to insert the meat portion. The modified burger was subsequently snap frozen to preserve the position of the introduced meat and facilitate sectioning. Cryo-sectioning and mounting were performed as described above for the other two burger samples.

### Optical Imaging

Tissue sections were optically scanned with a resolution of 4000 dpi before further preparation using a Canon CanoScan 9000F Mark II flatbed scanner with the VueScan 9.7.51 software (Hamrick Software; http://www.hamrick.com).

### Matrix Application

DHB (15 mg/mL in 70% MeOH with 0.2% TFA) was also sprayed using an HTX M3+ sprayer (HTX Imaging LLC, Carrboro, NC, USA) for detecting lipids and metabolites in positive ion mode. The following settings were used: 12 layers of the matrix were applied at a nozzle temperature of 60 °C, a velocity of 1200 mm/min, a flow rate of 120 μL/min, CC pattern, and a drying time of 30 s. NOR (7 mg/mL in 66% CHCl_3_ and 33% MeOH) was sprayed using HTX M3+ to analyze the distribution of small metabolites in negative ion mode as described elsewhere. The following settings were used: 15 layers of the matrix were applied at a nozzle temperature of 30 °C, a velocity of 1200 mm/min, a flow rate of 120 μL/min, CC pattern, and a drying time of 30 s.

### MALDI-MS Imaging

Samples were analyzed on a Bruker MALDI-2 timsTOF flex instrument (Bruker Daltonik, Bremen, Germany). The instrument was operated in reflectron mode with a mass range of *m*/*z* 100–1000 for both polarities. The data were acquired with 25 laser shots per pixel at a frequency of 1000 Hz. The transfer settings for positive ion lipid imaging were as follows: funnel 1 RF = 300 Vpp, funnel 2 RF = 400 Vpp, multipole RF = 700 Vpp, and collision RF = 1500 Vpp. The transfer settings for negative ion metabolite/lipid imaging were as follows: funnel 1 RF = 100 Vpp, funnel 2 RF = 100 Vpp, multipole RF = 200 Vpp, and collision RF = 650 Vpp. Focus pretime-of-flight (TOF) transfer time was set at 80 μs, and prepulse storage was set at 12 μs. The quadrupole ion energy was 5.0 eV, the collision cell energy was 10.0, and the spatial resolution was 50 × 50 μm.

### DESI-MS Imaging

Analysis was performed on a Waters Xevo G2-XS mass spectrometer equipped with a DESI source with a DESI XS high-performance spray emitter (Waters Corporation, Manchester, UK). Before each measurement, line scans were performed on a glass slide coated with sodium formate, and an average spectrum was generated for calibration (<0.5 ppm). The instrument was operated in positive ion mode, the spray voltage was 0.7 kV, the nitrogen gas pressure was 18 psi, and the capillary temperature was 150 °C. The spray solvent, MeOH/H_2_O (98:2, v/v), was directed onto the sample at an angle of 75° and a flow rate of 3 μL/min. Images were acquired at a pixel size of 50 × 50 μm with a scan rate of 200 μm/sec, which resulted in a scan time of 0.236 s/pixel. The images were generated using the HDImaging 1.5 software (Waters Corporation).

### Data Analysis and Machine Learning Model

For this study, a comprehensive data preprocessing approach was implemented in Python to process and analyze all of the MSI data. It is important to note that the data from the DESI and MALDI-MSI techniques were not combined in the development of the ML models in this Article. Initially, the data were converted from vendor data files into the imzML format. The data were converted into a continuous format and downbinned to a bin size of 0.05 u, a decision based on a balance between mass resolution and computational efficiency, to manage the data size effectively for analysis. Utilizing the pyimzML library,^19^ the MSI data were analyzed to extract pixel coordinates and intensity values across the mass-to-charge (*m*/*z*) range, resulting in a data cube where each *x*,*y* pixel contains a mass spectrum extending into a third dimension. The average mass spectrum of each imzML file was calculated and exported, and a peak-picking algorithm was employed to create a peak list with their corresponding full width at half-maximum (fwhm) using mMass. This list facilitated the calculation of peak boundaries, enabling the integration of peak areas by summing intensities within specified *m*/*z* ranges for each peak and thus transforming the raw data into a processed data set with defined peak intensities.

For the detection of adulteration, the peak picked data were loaded and dimensionality reduced using UMAP (metric = cosine, *n*_components = 3) and visualized as an RGB image by setting each reduced dimension into a range from 0 to 255 and overlaying them. A spatially aware, edge preserving algorithm was used to assist in the following processing step, as it was previously shown to benefit clustering.^20^ Finally, a *k*-means algorithm from the scikit-learn python library was used to cluster each pixel, and the image was reconstructed by assigning a different color for each cluster The number of clusters was determined visually based on a priori knowledge of the sample and set to 5. Finally, a random forest algorithm from scikit-learn was trained using the labels of each pixel, and the feature importance was used to investigate important peaks.

Each data set median was normalized after loading for the ingredient classification^21^ and then standardized using Z-score as final preprocessing before applying principal component analysis using the scikit-learn python library^22^ and keeping 10 principal components with >80% explained variance. Following this, manual segmentation was performed to separate the sample from the background for each analyte by picking pixels that held a principal component score above a manually determined threshold (Figures S1–3). Subsequently, Uniform Manifold Approximation and Projection (UMAP)^23^ was performed on the sample pixels of the ingredients by training a UMAP model (*n*_neighbors = 15, min distance = 0.1, *n*_components = 2, metric = cosine^24^), which was later used for metric learning. The pixels from the burger sample were fitted into the UMAP model, and the embedding was plotted. Finally, the original burger image was reconstructed by selecting for each vegetable burger pixel, and we found the nearest vegetable (carrot, pea, etc.) and visualized it in the same color. This was performed on both DESI and MALDI-MSI data sets.

A similar approach was used for the different burger classifications, but instead of adding each ingredient, different burgers were added. After segmentation to remove the background pixels, the data were used to train a Random Forest model, and the ion images of the top features were plotted to see the distribution of the important feature. This is in contrast to the use of semisupervised UMAP. This allowed for a quick examination of the distribution of peaks that were unique for each burger or common to all burgers.

### Tandem Mass Spectrometry

The *m*/*z* values selected for MS/MS analysis were those identified as significant by the classification model or those exhibiting unique distributions in the ingredients or burgers. A high-resolution Q Exactive HF Hybrid Quadrupole-Orbitrap (Thermo Fisher Scientific GmbH, Bremen, Germany) instrument coupled with an ESI injector (Spectroglyph, LLC, Kennewick, WA, USA) was employed for targeted tandem MS analysis. Sample preparation involved collecting shavings or trimmed samples during cryosectioning in a plastic vial. 200 mg of sample was combined with 1 mL of methanol and glass beads, then homogenized using a miniature bead beater (SUNON, MagLev, China) for two 20 s cycles. After centrifugation at 900 rpm for 10 min, the supernatant was transferred to a fresh vial and filtered through a 0.45 μm syringe filter to prevent instrument clogging. The filtered sample was then diluted 1:10 in 70% methanol (100 μL sample/900 mL methanol).

Samples were introduced via direct infusion as previously described.^25^ The mass spectrometer was operated in positive ion mode with a mass range of *m*/*z* 100–1000 and a mass resolution of 120 000. Maximum injection times were set to 50 ms for full MS and 2000 ms for MS/MS. The normalized collision energy ranged from 10 to 40 manufacturer units, with an isolation window of ±0.7 Da. The spray voltage was 3.4 kV, and the capillary temperature was maintained at 25 °C. Samples were injected at a rate of 2 μL per minute. MS/MS was performed using a high-energy collision dissociation cell.

The *m*/*z* values considered most important by the trained model were selected for ion image generation and identification. The MSI data set was imported to SCiLS Lab software 2024a (Bruker Daltonik, Bremen, Germany) to visualize the distribution of important features and create ion images. The *m*/*z* values corresponding to the background or MALDI-matrix were manually excluded while creating ion images.

## Results and Discussion

### MSI of Vegetables and Burgers

**Figure 1:**
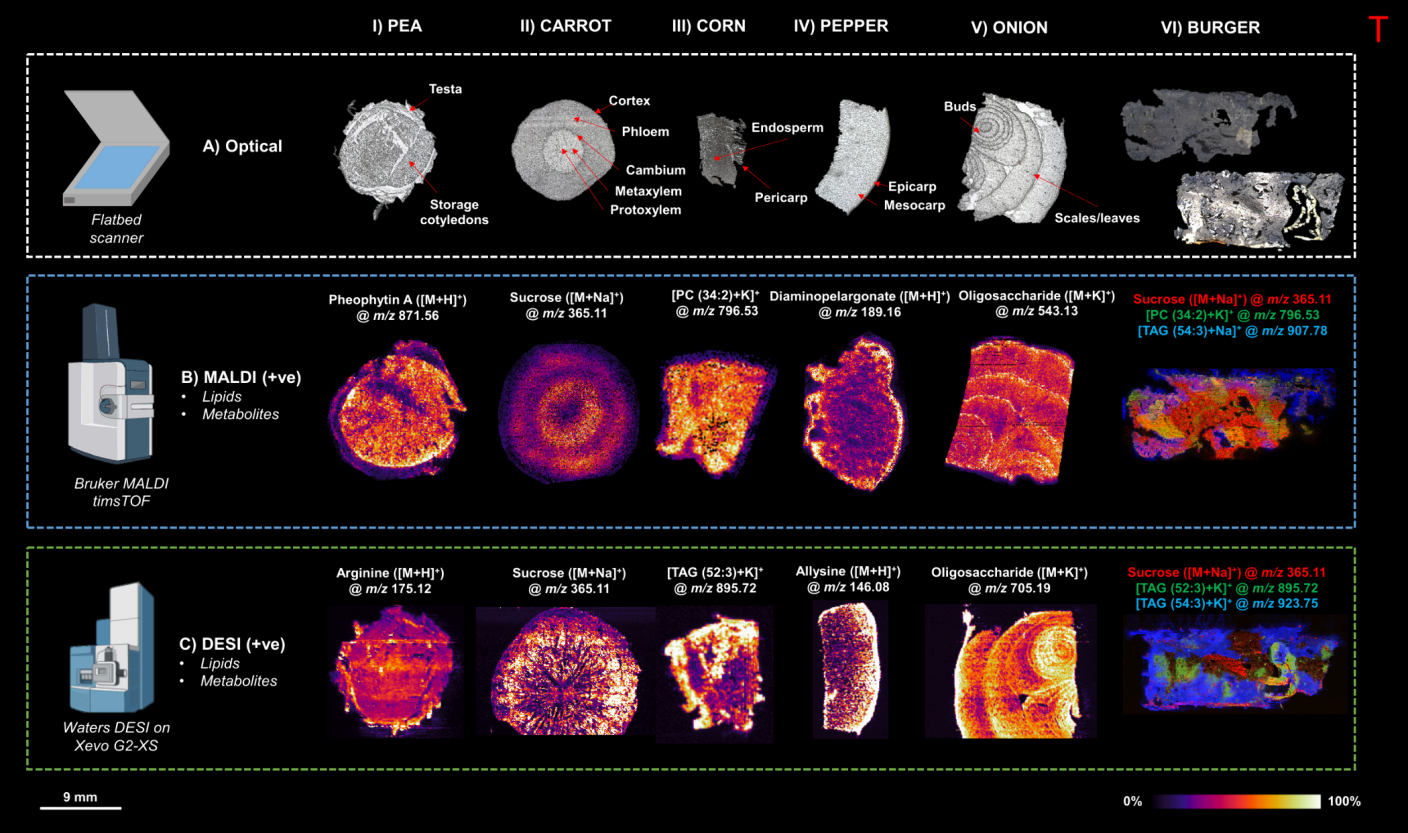
Comparison of different sample preparation methods and MSI techniques. (A) Optical images of annotated vegetable sections. (B) MALDI-MS images of samples prepared with DHB in positive ion mode and (C) DESI-MS images in positive ion mode (all images acquired at a spatial resolution of 50 × 50 μm and normalized with TIC).

Adulteration in food products has been detected in various forms, including the substitution of cheaper ingredients, incorrect labeling of components, and the presence of undeclared animal- or plant-based proteins. Analytical techniques commonly used to detect the composition of food materials and potential adulteration include PCR (polymerase chain reaction), LC-MS, and hyperspectral imaging.^26−28^ In this study, the data sets obtained from mass spectrometry imaging of burgers and their ingredients have been utilized to create machine learning models with different goals. The use of MALDI and DESI MSI data sets in this study has demonstrated the complementarity of these two techniques in detecting a wide range of compounds. It also highlights the effectiveness of the machine learning models that can be applied to data from both techniques. MALDI matrices enable the detection of a broad spectrum of metabolites and lipids, making it crucial for the model to work effectively with these compounds. The advantages of employing DESI are that it operates under ambient conditions and can eliminate matrix peaks, thus detecting low molecular weight compounds. Performing MALDI and DESI-MSI on vegetables enabled the distribution of several metabolites and lipids that were confirmed by tandem MS (Table S1). Figure 1 shows the distribution of some of those compounds in a vegetable burger along with their constituent vegetables. It illustrates the complementarity of two techniques used to study the distribution of different classes of molecules in vegetables and burgers. MALDI-MSI images show the distribution of pheophytin A [M + H]^+^ at *m*/*z* 871.56 in pea, disaccharide sucrose in the sodiated form ([M + Na]^+^) at *m*/*z* 365.11 in carrot, [PC (34:2) + K]^+^ in corn, diaminopelargonate ([M + H]^+^) at *m*/*z* 189.16, and a potassiated oligosaccharide at *m*/*z* 543.13. The RGB (red, green, and blue) image of the burger shows the distribution of sodiated sucrose, PC (34:2), and triacylglyceride [TAG (54:3)] at *m*/*z* 907.78. While sucrose and PC are also visualized in carrot and corn, respectively, the TAG is specific to burger in the regions where other ingredients such as vegetable oil, flour, and emulsifiers are expected.

DESI-MSI unveiled the distribution of arginine at *m*/*z* 175.12 in pea, sodiated sucrose at *m*/*z* 365.11 in carrot, TAG ([52:3] + K^+^) at *m*/*z* 895.72 in corn, allysine ([M + H]) at *m*/*z* 146.08, and potassiated oligosaccharide at *m*/*z* 705.19 in onion. The RGB image of the burgers shows sodiated sucrose and TAGs ([52:3] + K^+^) and ([54:3] + K^+^). Similar to MALDI-MSI, TAG ([54:3] + K^+^) is expected to come from ingredients other than vegetables.

### Machine Learning Model of a Modified Burger

**Figure 2:**
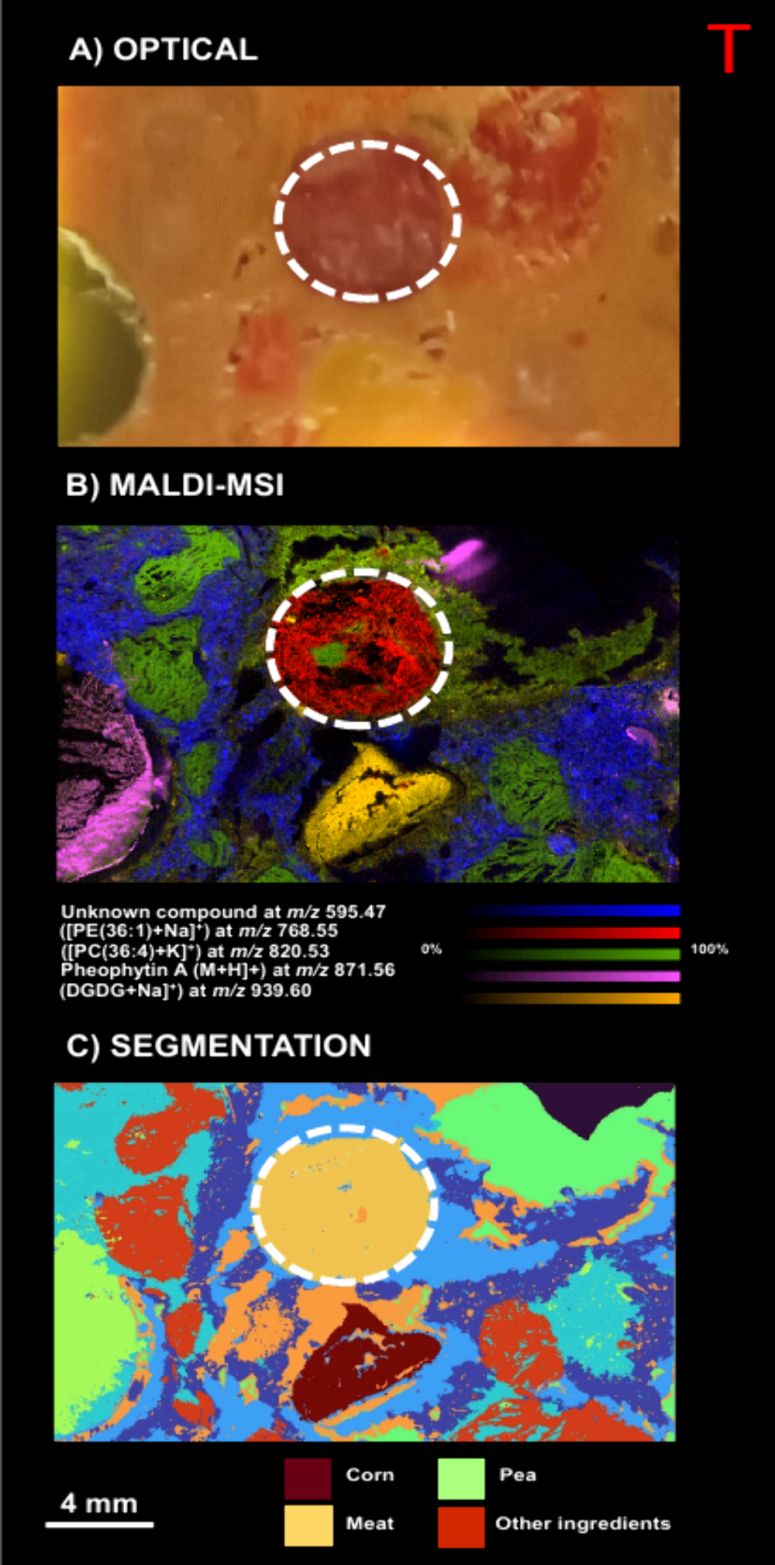
Discrimination of the modified vegetable burger. (A) Block face optical image of the modified vegetable burger (the added meat is indicated by the dashed circle). (B) MALDI-MS image showing the distribution of features of importance as determined by the machine learning model. The intensity scalebars in the bottom right belong exclusively to the MALDI-MSI panel. (C) Segmentation via a *k*-means algorithm showing each cluster as a unique solid color.

The goal of the first machine learning approach was to see whether addition could be detected. The MALDI data set was peak picked and loaded into a Pandas DataFrame, where it was dimensionality reduced using UMAP to three dimensions, similar to an RGB image. It was previously shown that applying a spatially aware edge-preserving image filter is beneficial to consequent clustering algorithms. The guided filter uses a guide image to smooth the target image. In the absence of a specialized guide image, the target image can also be used, which will successfully smooth the target image while preserving the edges. After this smoothing step, a *k*-means clustering algorithm was applied on the images, and a unique color was given to each cluster label, allowing for visualization (Figure 2C). The number of clusters was determined as five by user input based on a priori knowledge and the spatial homogeneity of the clusters. Following this, a random forest classifier model was trained to derive the feature importance, meaning that peaks that were deemed important by the model were able to distinguish between these different clusters. This unsupervised approach allows for quick detection and localization of important marker peaks and also the use of the model for future adulteration detection. These final peaks were manually targeted with tandem MS. The optical image of the block face (Figure 2A) shows the vegetable burger with some of the ingredients embedded within the patty, where the core of meat is indicated by the dashed circle. The corresponding MALDI-MS image (Figure 2B) shows the distribution of compounds that are specific to different areas, such as the meat core at *m*/*z* 768.58 (putatively identified as a PE (36:1) + Na] ^+^), and a piece of pea in the patty showed the distribution of pheophytin A at *m*/*z* 871.56. A piece of corn in the patty was defined by digalactosyldiacylglycerol [DGDG + Na] at *m*/*z* 939.60. The vegetable patty was defined by [PC (36:4) + K] ^+^) *m*/*z* 820.52 and unknown compound *m*/*z* 595.47 localized in the binding medium, which could be flour, oil, or other ingredients. The overlay of all these molecular species shows how they colocalize.

### Vegetable Burger Ingredient Classification

**Figure 3:**
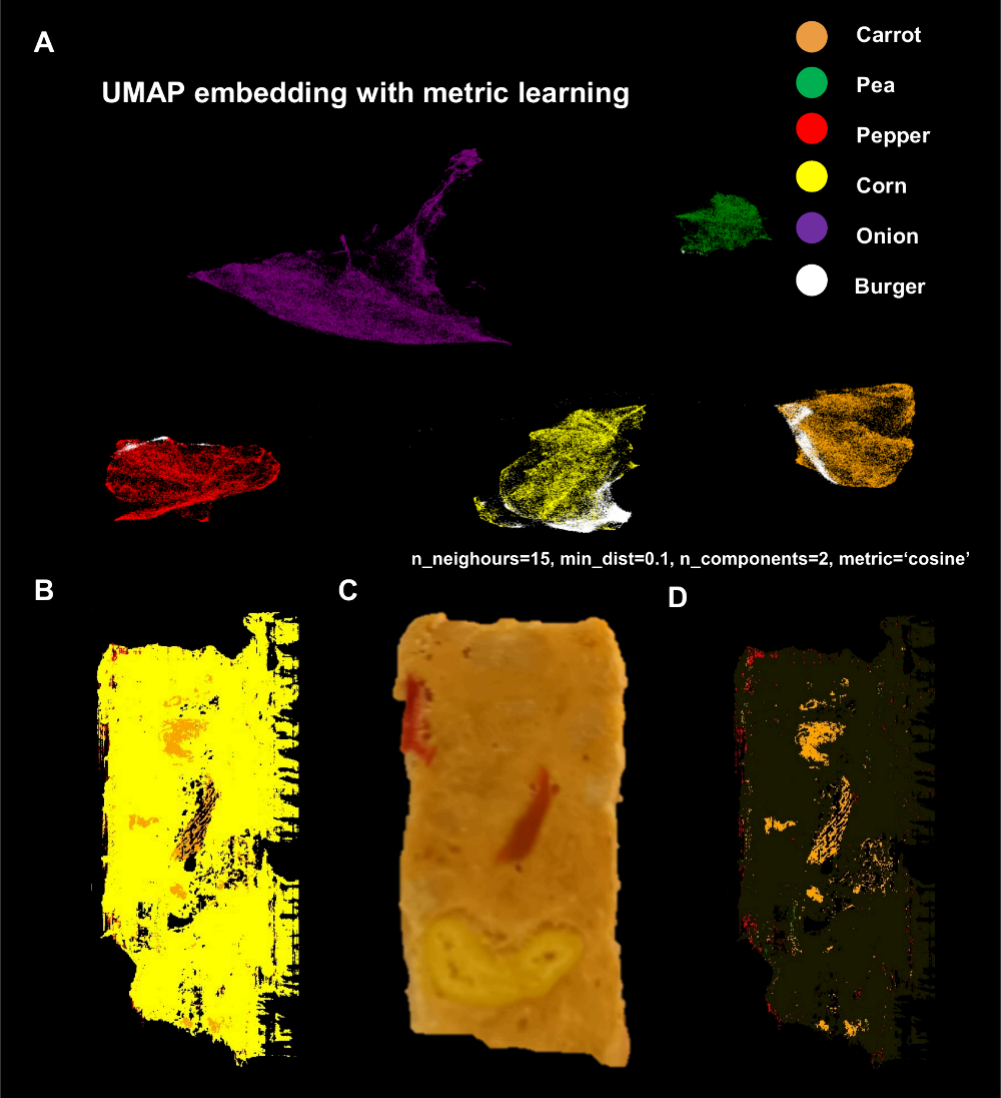
Classification of vegetable burger ingredients using DESI-MSI data. (A) Semisupervised UMAP embedding of sample pixels, with each ingredient forming discrete clusters. Using UMAP, vegetable burger pixels were classified among the ingredient clusters based on chemical similarity. (B) Reconstructed classification image based on the nearest ingredient for each vegetable burger pixel. (C) Optical image of the vegetable burger. (D) Reconstructed classification image, with corn made semitransparent for easier visualization of carrot and pepper.

It is expected that pixels corresponding to similar ingredients would have similar chemical profiles. Based on this hypothesis, a UMAP model was trained on the labeled sample pixels of each ingredient to for both MALDI (Figure S4) and DESI data sets to create a supervised embedding, and a classifier was built on that new space. As seen in Figure 3A, the embedding was able to separate each ingredient in its own cluster, showing that pixels of the same ingredients carry similar chemical information. The unlabeled pixels from the vegetable burger image were transformed into UMAP space. This process separated the pixels into groups corresponding to their ingredient labels based on molecular similarity. The burger pixels were distributed among different ingredient clusters, showing that certain pixels of the burger have high similarity to certain ingredients (Figure 3A). Based on the UMAP classification, the color of the nearest ingredient pixel for each burger pixel was selected. This allowed the reconstruction of the original vegetable burger image colored with classified ingredients (Figure 3B). From the optical image (Figure 3C), three ingredients can be distinguished: corn, carrot, and pepper, and UMAP classification succeeds in classifying all of them. Corn takes a significant percentage of the classified pixels, and this can be attributed to the preparation process of the burgers, which includes cooking. Among the individual sample ingredients tested, only the corn was cooked, while the others remained raw. This cooking process may have led to similar molecular profiles between the binding medium of the burger and the corn, potentially explaining why the model classified them together. The binding medium of the burger is predominantly soy and wheat, and these ingredients share a similar biological function in storing sugars with corn. As a result, it is hypothesized that the binding medium was assigned to corn due to the molecular similarities shared among corn, soy, and wheat. Finally, no pixel labels were given for the binding medium itself; therefore, the model was forced to classify it as one of the ingredient clusters. As explained above, corn shares the closest molecular profile to the binding medium, hence the result of the classification. Despite this limitation, this study shows that UMAP can be used in a semisupervised method to perform metric learning and classify components in a heterogeneous food matrix. The performance of the model can be further improved by more extensive optimization, but this was beyond the scope of this Article. Corn pixels were made semitransparent to improve the contrast of the other ingredients (Figure 3D).

### Differentiating between Different Burgers

**Figure 4:**
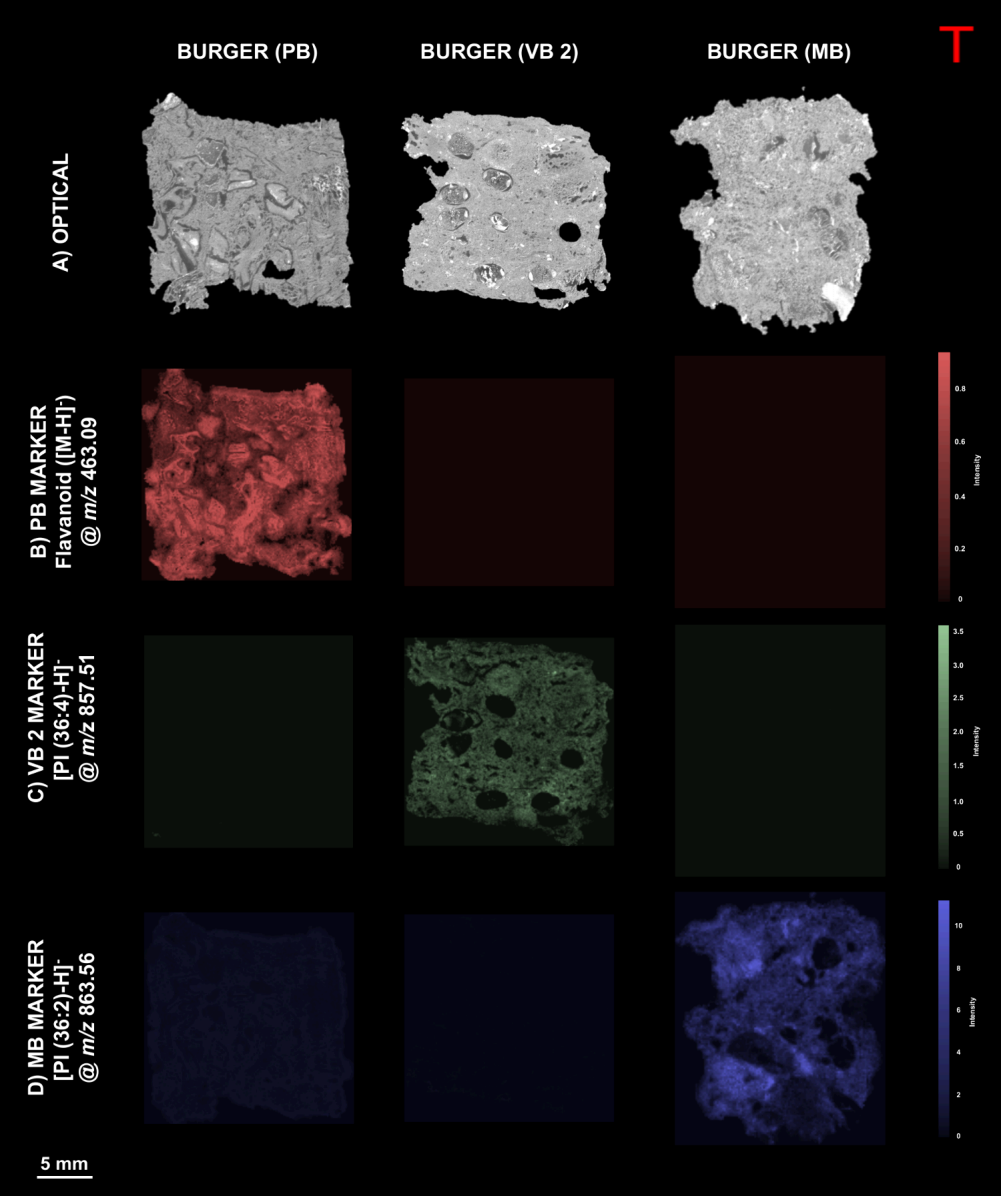
Comparison of different burgers. PB, VB2, and MB burgers based on a machine learning model. (A) Optical images of burger sections before matrix application. Molecular markers for (B) PB (flavonoid) at *m*/*z* 463.09, (C) VB 2 ([PI 36:4) – H]^−^) at *m*/*z* 857.51, and (D) MB ([PI 36:1) – H]^−^) at *m*/*z* 863.56.

Finally, it was of interest to see whether different burgers could also be differentiated from each other. MALDI data sets of VB 2, PB, and MB were analyzed with similar preprocessing steps as the ingredient classification. However, after the PCA segmentation, a random forest classifier model was trained instead on the different burgers, and this allowed the acquisition of the most important peaks for differentiating between the different burgers. Following this step, ion images of each highest-ranking feature were plotted, allowing for manual investigation on the spatial distribution of these peaks. MS/MS measurements were performed on the *m*/*z* values unique to each burger referred as markers in Figure 4. Burger PB has [PI (36:4) – H]^−^ at *m*/*z* 857.51, VB 2 at *m*/*z* 463.09 was putatively identified as a flavonoid [M – H]^−^, as this burger has vegetables and seeds as main constituents, and MB shows a marker [PI (36:1) – H]^−^ at *m*/*z* 863.56.

## Conclusions

The spatial distribution of molecules in food is crucial, as their arrangement significantly influences the experience of the consumer as well as the marketability of the product. MALDI and DESI-MSI were instrumental in elucidating the spatial distribution of compounds in vegetable ingredients and burger. This study successfully classified vegetable constituents of a burger using UMAP metric learning in a semisupervised way by employing MSI data sets. It could distinguish the addition of meat within the vegetable burger, vegetable ingredients in the pure vegetable burger, and different brands of burger. Additionally, the model was effective in working with both MALDI and DESI-MSI data sets. Future developments may include the integration of real-time analysis with production lines and the creation of spectral libraries for the rapid detection and imaging of ingredients within complex food matrices. This will ultimately lead to improved product transparency, quality control, process optimization, and consumer trust.

## Supporting Information Available

Principle component loadings and images, exact masses and MS/MS of detected compounds, and UMAP of MALDI-MSI data (PDF)
