# Supplementary material for: Mass Spectrometry Imaging for Spatial Ingredient Classification in Plant-Based Food
Source: J Am Soc Mass Spectrom. 2024 Dec 7;36(1):100–7. doi: 10.1021/jasms.4c00353 (PMC11697329; doi:10.1021/jasms.4c00353)
Supplement: Supplementary file 1 — js4c00353_si_001.pdf [file js4c00353_si_001.pdf]

Supporting Information

Mass spectrometry imaging for spatial ingredient classification in plant-based food

Mudita Vats[1], Bryn Flinders[1], Theodoros Visvikis[1], Corinna Dawid[2,3], Thomas F. Hofmann[2], Eva Cuypers[1], Ron M. A. Heeren[1,4*]

[1.] Maastricht MultiModal Molecular Imaging (M4I) Institute, Division of Imaging Mass Spectrometry, Maastricht University, Universiteitssingel 50, 6229 ER Maastricht, The Netherlands.

[2.] Chair of Food Chemistry and Molecular and Sensory Science, Technical University of Munich, Lise-Meitner-Str. 34, Freising 85354, Germany.

[3] Professorship for Functional Phytometabolomics, TUM School of Life Sciences, Technical University of Munich, Lise-Meitner-Str. 34, Freising 85354, Germany.

[4] Focus group Molecular Imaging of Cellular Metabolism, Institute for Advanced Studies, Technical University of Munich, Lichtenbergstraße 2a, 85748 Garching, Germany

Table of Contents

Figure S1. PC 1 and PC 2 loadings of DESI-MSI dataset 2

Figure S2. PC 3 loadings of DESI-MSI dataset 3

Table S1. Exact masses of compounds 4

Figure S4. UMAP of MALDI-MSI data 5

Figure S5. An example of a comparison between (A) MALDI and (B) DESI MSI spectra of burger sample in positive ion mode and (C) MALDI-MSI spectra of burger in negative ion mode…………… 6

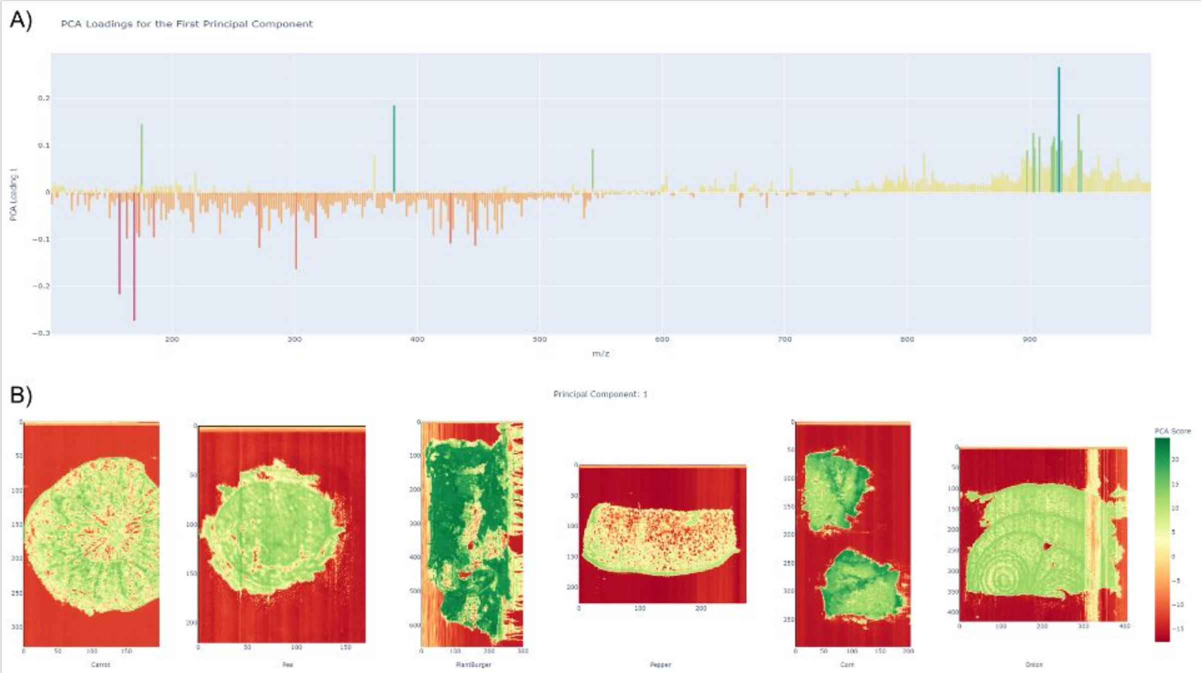

Figure S1. DESI -MSI dataset showing the first principle component of all samples. A) PC1 loadings of each *m/z* plotted as a spectrum. B) PC1 scores of each pixel visualized as the original images.

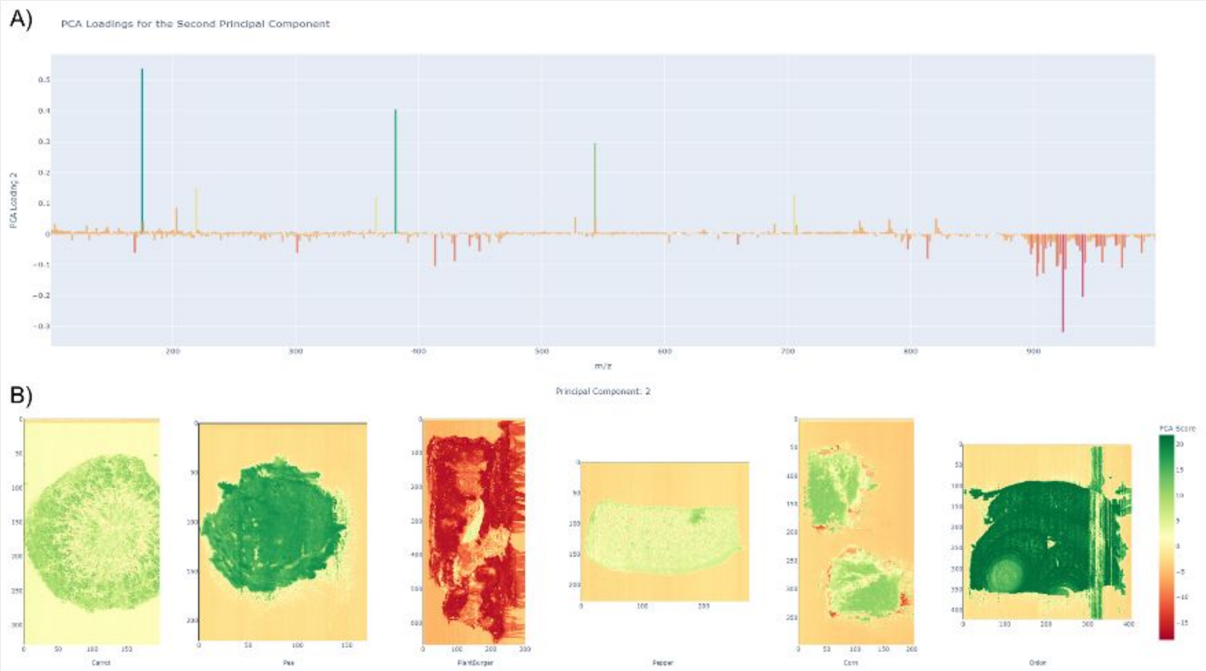

Figure S2. DESI -MSI dataset showing the second principle component of all samples. A) PC2 loadings of each *m/z* plotted as a spectrum. B) PC2 scores of each pixel visualized as the original images.

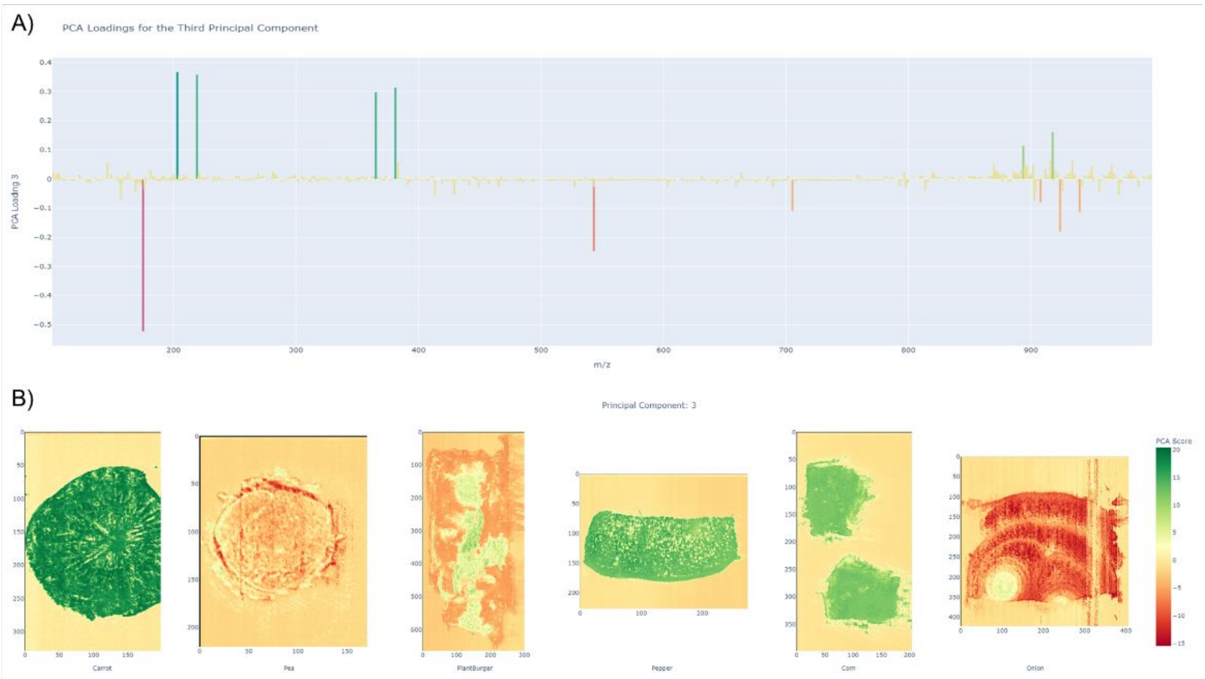

Figure S3. DESI -MSI dataset showing the third principle component of all samples. A) PC3 loadings of each *m/z* plotted as a spectrum. B) PC3 scores of each pixel visualized as the original images.

Table S1. Exact masses obtained from burgers and individual vegetables by ESI-MS and MS/MS.

Observed mass	***Theoretical mass***	***Formula***	***Error (ppm)***	***Identification***	***MS/MS framents (%)***	***NCE***
184.0737	184.0733	$C_5H_{15}NO_4P$	1.896	Phosphocholine	104 (100)	10
381.0795	381.0794	$C_{12}H_{22}O_{11}K$	0.42	Sucrose	219.0269 (30), 201.0163 (15)	35
758.5703	758.5694	$C_{42}H_{81}NO_8P$	1.119	$[PC (16:0\_18:2)+H]^+$	184.0735 (100), 496.3403 (2), 502.3299 (1)	20
784.5869	784.5851	$C_{44}H_{83}NO_8P$	2.356	$[PC (18:1\_18:2)+H]^+$	184.0736 (100), 520.3404 (1)	12
496.3404	496.3396	$C_{24}H_{51}NO_7P$	1.298	$[LPC (16:0)+H]^+$	104.1076 (80), 184.0736 (100), 478.3298 (10)	20
820.5255	820.5253	$C_{44}H_{80}NO_8PK$	0.203	$[PC (36:4)+K]^+$	86.0972 (90), 162.9558 (100), 599.5040 (20), 637.4598 (15), 761.4523 (50)	25
796.5262	796.5253	$C_{42}H_{80}NO_8PK$	1.051	$[PC (36:2)+K]^+$	737.4523 (100), 162.9559 (80), 86.0972 (60)	22
534.2963	534.2956	$C_{24}H_{50}NO_7PK$	1.24	$[LPC (16:0)+K]^+$	86.0972 (15), 104.1076 (100), 162.9559 (5) 475.2226 (60)	20
558.2962	588.2956	$C_{26}H_{50}NO_7PK$	1.025	$[LPC (18:2)+K]^+$	86.0972 (15), 104.1076 (100), 162.9559 (5) 499.2226 (60)	20
381.0794	381.0794	$C_{12}H_{22}O_{11}K$	0.106	$[Disaccharide+K]^+$	219.0269 (30), 201.0163 (15)	35
543.1323	543.1322	$C_{18}H3_2O_{16}K$	0.252	$[Trisaccharide+K]^+$	363.0690 (10), 381.0796 (100)	35
705.1853	705.185	$C_{22}H_{42}O_{21}K$	0.402	$[Oligosaccharide+K]^+$	363.0689 (30), 381.0796 (20), 525.1222 (10), 543.1328 (80)	25
867.2385	867.2378	$C_{30}H_{52}O_{26}K$	0.784	$[Oligosaccharide+K]^+$	363.0689 (10), 381.0795 (20), 525.1220 (30), 543.1324 (90), 705.1851 (100)	32
175.1192	175.119	$C_6H_{15}O_2N_4$	1.186	$[Arginine+H]^+$	60.0566 (60), 70.0660 (100), 116.0711 (70), 130.0978 (40), 158.0926 (20)	40
804.5526	804.5514	$C_{44}H_{80}NO_8PNa$	1.559	$[PC (36:4)+Na]^+$	86.0972 (50), 146.9819 (95), 599.5037 (30), 621.4856 (100), 745.4780 (30)	25
758.5706	758.5694	$C_{42}H_{81}NO_8P$	1.514	$[PC (16:0\_18:2)+H]^+$	184.0735 (100), 496.3403 (2), 502.3299 (1)	20
780.5525	780.5514	$C_{42}H_{80}NO_8PNa$	1.427	$[PC (34:2)+Na]^+$	86.0972 (50), 146.9819 (100), 184.0735 (50), 575.5038 (25), 597.4856 (60), 721.4780 (30)	25
782.5714	782.5694	$C_{44}H_{81}NO_8P$	2.516	$[PC (36:4)+H]^+$	184.0735 (100)	20
796.5265	796.5253	$C_{42}H_{80}NO_8PK$	1.427	$[PC (34:2)+K]^+$	86.0972 (80), 162.9558 (100), 575.5040 (10), 613.4812 (10), 737.4525 (50)	25
820.52565	820.5253	$C_{44}H_{80}NO_8PK$	1.495	$[PC (36:4)+K]^+$	86.0972 (80), 162.9558 (100), 599.5041 (15), 637.4809 (20), 761.4521 (50)	25

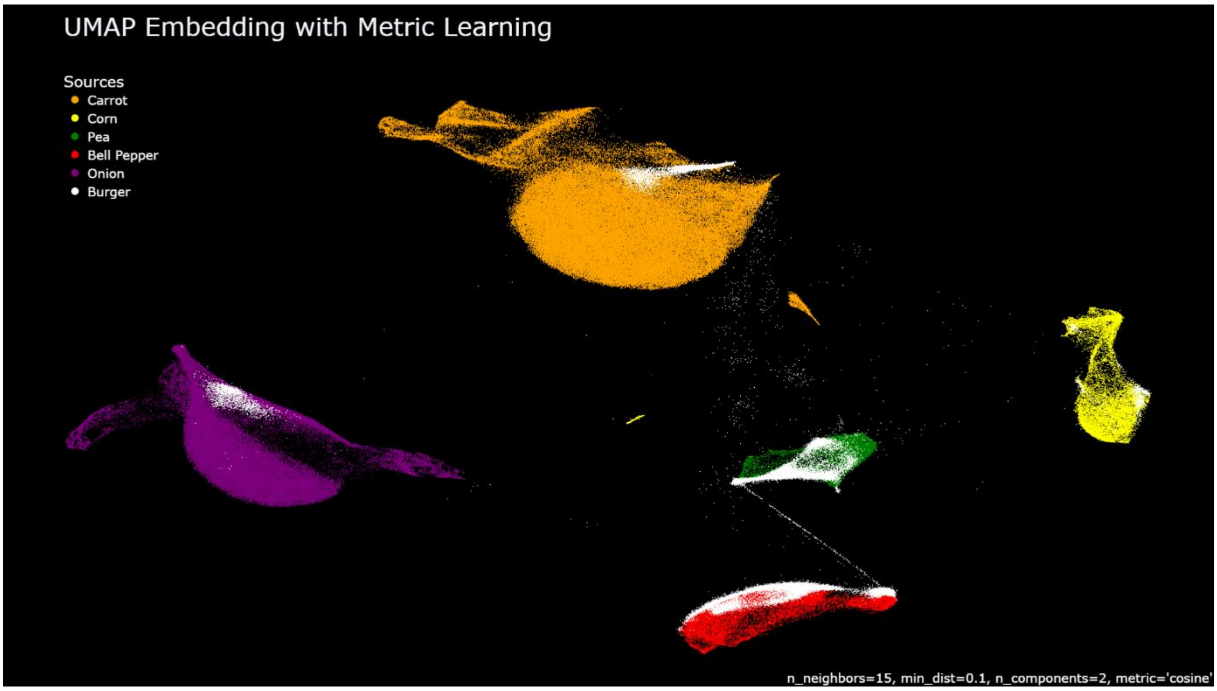

Figure S4. UMAP embedding with metric learning of MALDI-MSI dataset.

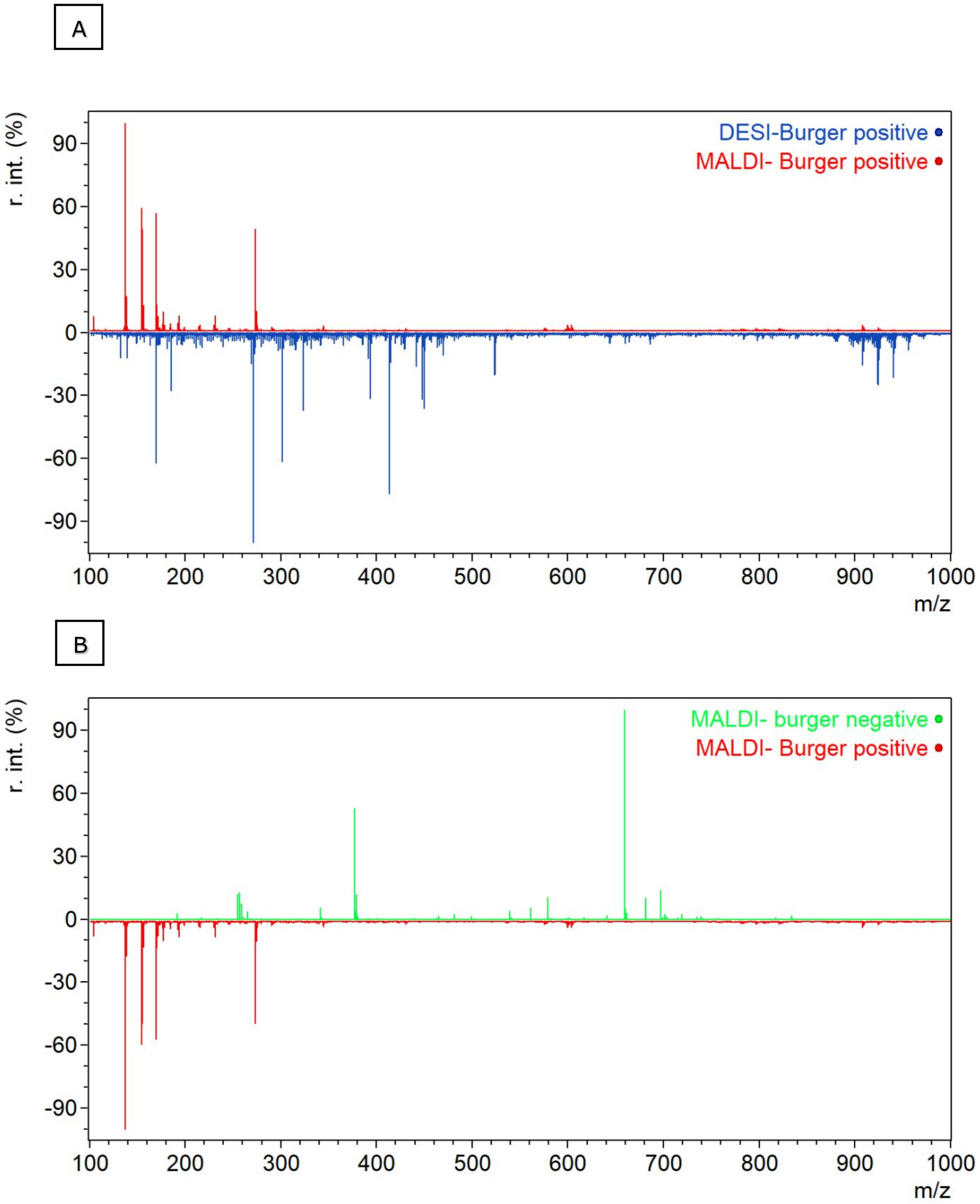

Figure S5. An example of a comparison between (A) MALDI and DESI MSI spectra of burger sample in positive ion mode and (B) MALDI-MSI spectra of burger in positive and negative ion mode.
